# Detection of *Bar* Transgenic Sugarcane with a Rapid and Visual Loop-Mediated Isothermal Amplification Assay

**DOI:** 10.3389/fpls.2016.00279

**Published:** 2016-03-08

**Authors:** Dinggang Zhou, Chunfeng Wang, Zhu Li, Yun Chen, Shiwu Gao, Jinlong Guo, Wenying Lu, Yachun Su, Liping Xu, Youxiong Que

**Affiliations:** Key Laboratory of Sugarcane Biology and Genetic Breeding, Fujian Agriculture and Forestry University, Ministry of AgricultureFuzhou, China

**Keywords:** genetically modified organism (GMO), transgenic sugarcane, *bar*, loop-mediated isothermal amplification (LAMP), PCR, detection

## Abstract

Genetic engineering offers an attractive alternative in sugarcane breeding for increasing cane and sugar yields as well as disease and insect resistance. *Bar* transgenic sugarcane employing the herbicide tolerance is a useful agronomical trait in weed control. In this study, a loop-mediated isothermal amplification (LAMP) assay for rapid detection of the *bar* gene in transgenic sugarcane has been developed and evaluated. A set of six primers was designed for LAMP-based amplification of the *bar* gene. The LAMP reaction conditions were optimized as follows: 5.25 mM of Mg^2+^, 6:1 ratio of inner vs. outer primer, and 6.0 U of *Bst* DNA polymerase in a reaction volume of 25.0 μL. The detection limit of the recombinant plasmid 1Ac0229 was as low as 10 copies in the developed LAMP, which was 10-fold higher sensitive than that of conventional PCR. In 100 putative transgenic lines, the *bar* gene was detected in 100/100 cases (100%) by LAMP and 97/100 cases (97%) by conventional PCR, respectively. In conclusion, the developed LAMP assay is visual, rapid, sensitive, reliable, and cost-effective for detection of the *bar* specific transgenic sugarcane.

## Introduction

Sugarcane (*Saccharum* spp.), a major crop of tropical and sub-tropical regions (Henry and Kole, 2010), is the highest yielding crop worldwide and accounts for ~80% of the sugar (sucrose) production in the world (Nayak et al., 2014) and around 92% of that in China (Luo et al., 2012). Sugarcane can also be used to produce ethanol as an attractive biofuel feedstock in recent years (Henry and Kole, 2010). Sugarcane belongs to the species of vegetative propagation, and most modern sugarcane varieties are only the sexual generations from wild plants, which suggests that sugarcane is of significant potential for further genetic improvement (Henry and Kole, 2010). However, it usually takes more than 10 years for traditional breeders to identify and release a new elite sugarcane variety (Berding and Roach, 1987; D'Hont et al., 1996) from a huge segregated population (Chen et al., 2011). Besides, the complex genome, narrow genetic base, poor fertility, and susceptibility to biotic and abiotic stresses of sugarcane also limit the traditional breeding (Suprasanna et al., 2011). Due to the fact that it can shorten the breeding period, reduce the cost and manifest a stable inheritance, genetic engineering becomes an efficient alternative and a useful tool for sugarcane improvement (Arencibia et al., 1999; Gaskell et al., 1999; Falco et al., 2000; Jain et al., 2007). It is particularly attractive for sugarcane because improved clones can be routinely multiplied and maintained by vegetative propagation (Falco et al., 2000). Compared with other crops, genetic engineering in sugarcane has the lowest security risk (level I; Chen et al., 2011; Zhou et al., 2014). Thus, genetic engineering holds promise for increasing cane and sugar yields as well as weed, disease, and insect resistance in sugarcane (Gilbert et al., 2005).

Controlling weeds is one of the most important tasks in sugarcane cultivation and management at the early growth stage. Unfortunately, owing to the lack of herbicide resistant genes in gene pool, just like other crop species, sugarcane is sensitive to the herbicide and thus it needs exogenous genes to improve its herbicide resistance. There has been a tremendous success in gene transfer from a wide variety of plant and non-plant sources to plant, including sugarcane (Leibbrandt and Snyman, 2003; Suprasanna et al., 2011). The development of herbicide-resistant crops was one of the first commercial applications of genetic engineering in plant breeding (Leibbrandt and Snyman, 2003). Genetically modified sugarcane resistant to herbicide, usually with transformation of the genes such as *bar* and *epsps* into sugarcane genome, has been reported (Gallo-Meagher and Irvine, 1996; Falco et al., 2000). Moreover, *bar* was also the most important and widely used selectable marker for genetic transformation (Bower and Birch, 1992; Fitch et al., 1995). A localized application of the phosphinothricin (PPT) solution on young leaves can be used for the *in vitro* confirmation of herbicide-resistant transformed plants (Bower and Birch, 1992; Fitch et al., 1995). However, a large candidate segregated population for selection and the relative heavy selection work together with the usual occurrence of phenotypic lag is still a significant investment in sugarcane breeding program (D'Hont et al., 1996; Zhou et al., 2014). Therefore, it is necessary to develop a rapid, stable and low-cost technique for the early detection of foreign gene in transgenic sugarcane, such as *bar* transgenic lines, for supervision and administration purposes.

To date, for genetically modified organism (GMO) detection, a multitude of developed methods were reported, and their number is increasing rapidly. These detection methods have been divide into protein-based and nucleic acid-based detection techniques (Dong et al., 2008; Morisset et al., 2008), such as enzyme-linked immunosorbent assay (ELISA), lateral flow strip, western blot, conventional PCR, competitive PCR, real-time PCR, Southern blot, and micro-array (Dong et al., 2008; Morisset et al., 2008). Though the methods based on conventional PCR and real-time PCR are the most wildly used laboratory techniques, there are some limitations in specific areas of application like on-site detection since it required expensive qualitative or quantitative PCR instrument (Morisset et al., 2008; Zhou et al., 2014). Furthermore, real-time quantitative PCR has the accuracy limits due to its exponential amplification nature (Morisset et al., 2008).

As the alternatives to PCR, several miniaturized analysis systems, specifically isothermal amplification reactions have been developed and introduced into the routine detection and GMO detection (Asiello and Baeumner, 2011; Zanoli and Spoto, 2012). The isothermal methods do not need thermal cycling and thus isothermal microsystems can outperform PCR in portable, battery-operated detection systems (Asiello and Baeumner, 2011; Zanoli and Spoto, 2012). The main isothermal methods include loop-mediated isothermal amplification (LAMP; Notomi et al., 2000), nucleic acid sequence-based amplification (NASBA; Mugasa et al., 2014), rolling circle amplification (RCA; Ali et al., 2014), helicase-dependent amplification (HDA; Barbieri et al., 2014), strand displacement amplification (SDA; Qiu et al., 2013), isothermal and chimeric primer-initiated amplification of nucleic acids (ICANs; Asiello and Baeumner, 2011), and signal-mediated amplification of RNA technology (SMART; Zanoli and Spoto, 2012). Among them, LAMP has been widely adopted and further developed for widespread clinical use (Lee et al., 2015; Nakano et al., 2015; Neeraja et al., 2015; Oriero et al., 2015), the diagnosis of infectious diseases (Mori and Notomi, 2009; Kinoshita et al., 2015; Mansour et al., 2015), rapid testing of food products (D'Agostino et al., 2015; Ferrara et al., 2015; Sun et al., 2015) and environmental samples (Niessen, 2015; Shi et al., 2015), and also for detecting exogenous genes in GMOs (Zhou et al., 2014; Liu et al., 2015; Feng et al., 2015; Huang et al., 2015; Singh et al., 2015; Wang et al., 2015; Zhang et al., 2016) in the past decade.

LAMP, which was originally invented by Notomi et al. (2000), can complete automatically looping, strand displacement and DNA synthesis using *Bst* DNA polymerase and four specific primers (two outer primers F3 and B3, along with two inner primers FIP and BIP, which specifically recognize six distinct regions of the target DNA sequence) and optional addition of two specific loop primers (LF and LB; Notomi et al., 2000, 2015; Morisset et al., 2008). The LAMP technique has been widely used for detecting specific target genes in GM crops (Morisset et al., 2008; Notomi et al., 2015). For GM detection by LAMP assay, the targets can be commonly employed promoters (such as *P-35S, P*-FMV; Fukuta et al., 2004; Randhawa et al., 2013), marker genes (such as *npt*II and *uidA*; Randhawa et al., 2013), specific genes (such as *cry1Ac*; Li et al., 2013; Zhou et al., 2014; Singh et al., 2015, *cry2Ab* and *cp4-epsps;* Li et al., 2013; Zhou et al., 2014; Singh et al., 2015), and specific transgenic event [GM rice KMD1 and TT51-1 (Chen et al., 2012), GM maize T25 (Xu et al., 2013), and GM wheat B73-6-1 (Cheng et al., 2014)]. Fukuta et al. reported the detection of the *CaMV35S* promoter in Roundup-Ready soybean, which introduced the LAMP method to the GM screening field for the first time (Fukuta et al., 2004). Randhawa et al. developed LAMP system for screening GMOs using specific primers which recognized *P-35S, P*-FMV, aadA, *nptII*, and *uidA* (Randhawa et al., 2013). Zhou et al. optimized a LAMP system to detect *cry1Ac* transgenic sugarcane, with the sensitivity of 10~100 times higher than conventional PCR (Zhou et al., 2014). Singh et al. developed the LAMP assays for commonly employed transgenic elements of *cry1Ac, cry2Ab2*, and *cp4-epsps*, and confirmed their specificity (Singh et al., 2015). However, there is still no report on using LAMP to detect *bar*-transgenic sugarcane.

In the present study, a time-efficient, user-friendly, sensitive, accurate and robust visual LAMP detection technique for *bar* transgenic sugarcane has been developed. The specificity and sensitivity of the primers in the LAMP reactions were evaluated. The results indicated that the developed LAMP assay is more sensitive than conventional PCR and could be used for GM detection in the field. The LAMP technique developed here would facilitate *bar*-specific screening to check for GM sugarcane, as well as to monitor *bar*-specific GM contamination in the field.

## Materials and methods

### Plant materials and DNA extraction

Six non-transgenic sugarcane (*Saccharum* spp.) lines including three modern sugarcane cultivars (*S*. spp. Hybrids) FN15, ROC22 and ROC10; One *S. officinarum* Badila; Two *S*. wild species *S. spontaneum* 82-114 and *S. robustum* 57NG208, together with two *bar* transgenic sugarcane lines (a1 and 16k-2 from host cultivar FN15 and ROC22, respectively) and 100 putative *bar* transgenic sugarcane lines were used as the test materials. All the plant materials are provided by the Key Lab of Sugarcane Biology and Genetic Breeding, Fujian Agriculture and Forestry University, Ministry of Agriculture, China. The genomic DNA (gDNA) of all these cultivars/lines was extracted using the modified CTAB protocol reported by Aljanabi et al. (1999), employing polyvinyl pyrrolidone (PVP) to remove polyphenols, and using high salt concentrations to remove polysaccharides, along with an extended RNase treatment and a phenol-chloroform extraction. The DNA quality was assessed by agarose gel electrophoresis (AGE) and the DNA purity was determined by calculating the A_260_/A_280_ ratio using NanoVue Plus™ (GE, New Jersey, USA). The final DNA concentration was adjusted to 25 ng·μL^−1^.

Plasmid pGcry1Ac0229 (1Ac0229; Figure S1) was constructed and used as a positive control to optimize the LAMP reaction. Plasmid 1Ac0229 was constructed from pGreen II 0229 (which was obtained from the John Innes Centre in England, and containing the *bar* gene) and cassette *35s*-*cry1Ac-nos*, which were digested with *Eco*R I and *Hin*d III, and linked by T_4_-DNA ligase (Figure S1). The plasmid DNA was extracted from the transformed *Escherichia coli* DH5a strain by Plasmid Mini Kit I (Bio-tek Co., Ltd., Beijing, China), and identified by PCR with a 140 bp amplicon as shown in Figure S2, indicating the successful incorporation of the *bar* gene into the plasmid. In addition, sequencing (Invitrogen, Life Technologies Co., Ltd., Shanghai, China) result also showed that 1Ac0229 had been successfully recombined with the *bar* gene (data not shown). The concentration was determined by GE NanoVue Plus™ and the original copy number of this plasmid was adjusted to 1.0 × 10^9^ per μL.

### Primer design

LAMP primers were designed on the basis of the 552 bp *bar* gene (GenBank accession number EU048867.1) using the Primer Explorer 4.0 software (http://primerexplorer.jp/e/, Eiken Chemical, ToKyo, Japan). A set of six primers comprising two outer primers (F3 and B3), two inner primers (FIP and BIP), and two loop primers (LF and LB), which recognize a total of eight distinct regions of *bar* gene were designed. Primer design chart and primer sequences were shown in Figure S3 and Table S1, respectively. One set of primers (*bar*-1F: 5′-TTTCGGTGACGGGCAGGAC-3′, *bar*-1R: 5′-GCACGAGGCGCTCGGATAT-3′) with the amplification product of 140 bp was used for the specific detection of *bar* gene by PCR. All the LAMP primers at HPLC grade, were synthesized by TaKaRa Biotechnology Co., Ltd., Dalian, China.

### Reaction mixture for LAMP

The initial condition of the LAMP reaction was adopted from Zhou et al. (2014). The initial LAMP was carried out in a 25.0 μL mixture containing 2.5 μL 10 × ThermoPol Reaction Buffer [New England Biolabs, Massachusetts, USA, including 20.0 mM Tris-HCl (pH 8.8), 10.0 mM KCl, 2.0 mM MgSO_4_, 10.0 mM (NH_4_)_2_SO_4_, 0.1% Triton X-100], 3.75 mM MgSO_4_ (50.0 mM, Sigma-Aldrich Inc., St. Louis, USA), 0.8 μM each FIP and BIP, 0.2 μM each F3 and B3, 0.4 μM each LF and LB, 1.4 mM dNTPs (10.0 mM, TaKaRa Biotechnology Co., Ltd., Dalian, China), 8.0 U *Bst* DNA polymerase large fragment (New England Biolabs, Massachusetts, USA) and a specified amount of sugarcane genomic DNA or plasmid 1Ac0229.

The mixture was incubated at 65°C for 60 min, followed by heating at 80°C for 5 min to inactivate the *Bst* DNA enzyme and terminate the reaction. Products were then kept at 4°C.

Real-time LAMP was introduced to optimize the LAMP. Real-time LAMP reaction assays were performed on an ABI 7500 System (Applied Biosystems, Foster, USA) using final volumes of 25.0 μL, consisting of 0.5 × SYBR Green I (Bio-tek Co., Ltd., Beijing, China) except the LAMP reaction mixture mentioned above with the following conditions in isotherm model and FAM: 65°C for 70 min.

### Optimization of LAMP

Genomic DNA from non-transgenic cultivars FN15 was used as the negative control and ddH_2_O sterilized by filtration with 0.2 μm filter membrane (Millipore, Carrigtwohill, Co., Ltd. Cork, Ireland) after 121°C high-pressure steam sterilization for 1 h was used as a blank control, while plasmid 1Ac0229 was used as a positive control.

Based on the initial conditions of the LAMP reaction adopted from Zhou et al. (2014), five Mg^2+^ concentrations (4.75, 5.00, 5.25, 5.50, and 5.75 mM), four *Bst* DNA polymerase concentrations (2.0, 4.0, 6.0, and 8.0 U) and four concentration ratios between inner to outer primers (2:1, 4:1, 6:1, and 8:1) were tested in 25.0 μL reaction system, while the concentrations of all the other components remained constant respectively.

### Analysis for LAMP products

Following amplification by the LAMP method, the products were detected by the addition of 2.0 μL 1000 × SYBR Green I (Bio-tek Co., Ltd., Beijing, China) to the cap center of the tubes as reported by Zhou et al. (2014). Samples that turned yellowish-green were considered positive, while those that remained orange were assumed to be negative (Guan et al., 2010).

For real-time LAMP, the amplification curve is achieved with the reaction progress. The LAMP amplicon was detected as a value of fluorescence (delta Rn) in real-time using an increase in fluorescence intensity from the intercalating dye SYBR Green I.

### PCR reaction

For PCR, the reactions were performed using final volumes of 25.0 μL, including 12.5 μL 2 × Ex-Taq premix (Mg^2+^ Plus), 0.4 μM each primer (TaKaRa Biotechnology Co., Ltd., Dalian, China), and 1.0 μL template DNA (Zhou et al., 2014). All of the amplifications were performed using a thermal cycler (ABI Veriti 96, ABI, USA) with the following parameters: one step of 5 min at 94°C, 30 cycles of 30 s at 94°C, 30 s at 57°C, 20 s at 72°C, and one step of 7 min at 72°C. All PCR products were detected by electrophoresis on a 2.0% (w/v) agarose gel containing EB (0.5 μg·mL^−1^) in 1 × TAE buffer (pH 8.0) at 100 V for 1 h and were visualized under UV light.

### Sensitivity comparison between LAMP and conventional PCR

The sensitivity was compared between the optimized LAMP and conventional PCR with templates of 10-fold serial dilutions of plasmid 1Ac0229, of which the original copy number was adjusted to 1 × 10^9^ per μL.

### Specificity of LAMP

Specificity of the optimized LAMP system was verified using 50 ng of the following eight sugarcane genotypes' gDNA as template, including *bar* transgenic sugarcane lines a1 and 16k-2, modern sugarcane cultivars *S*. spp. hybrids FN15, ROC22 and ROC10, together with one *S. officinarum* Badila, and two wild species: *S. spontaneum* 82-114 and *S. robustum* 57NG208. *APRT* gene was selected as the internal positive control (Xue et al., 2014).

### Putative *bar* transgenic lines detected by LAMP and conventional PCR

In order to assess the reliability of the LAMP reaction system developed in this study, 100 putative *bar* sugarcane lines resistant to PPT were randomly collected from an experimental station at Fujian Agriculture and Forestry University. These *bar* transgenic sugarcane lines were named in the order of p1 ~ p100. The youngest fully expanded leaf, namely +1 leaf, with a visible dewlap (the collar between the leaf blade and sheath), was collected from each putative *bar* transgenic line. Then, 0.5 g fresh leaves of each sample were used for gDNA extraction. We selected *APRT* gene as the internal positive control to ensure that the gDNA quality is ok (Xue et al., 2014). In parallel, all of the 100 putative *bar* transgenic sugarcane lines were analyzed by both the developed LAMP assay and conventional PCR with a gDNA concentration of 50.0 ng·μL^−1^. Three biological replicates and three technical replicates were conducted for each sample.

In order to further detect the reliability of the LAMP assay in putative bar transgenic sugarcane, quantitative SYBR Green real-time PCR was applied to estimate the copy number of the *bar* gene (Primers are: *bar*-qF: CTTCAGCAGGTGGGTGTA, *bar*-qR: CAACGCCTACGACTGGAC; Xue et al., 2014). In addition, for those lines positive in LAMP assay but negative in PCR detection, quick test strip kit (QuickStix™ Kit for PAT/bar, Envirologix, Inc., USA) was used to measure bar protein expression according to the manufacturer's instructions.

## Results

### Optimization of LAMP reaction

**Figure 1:**
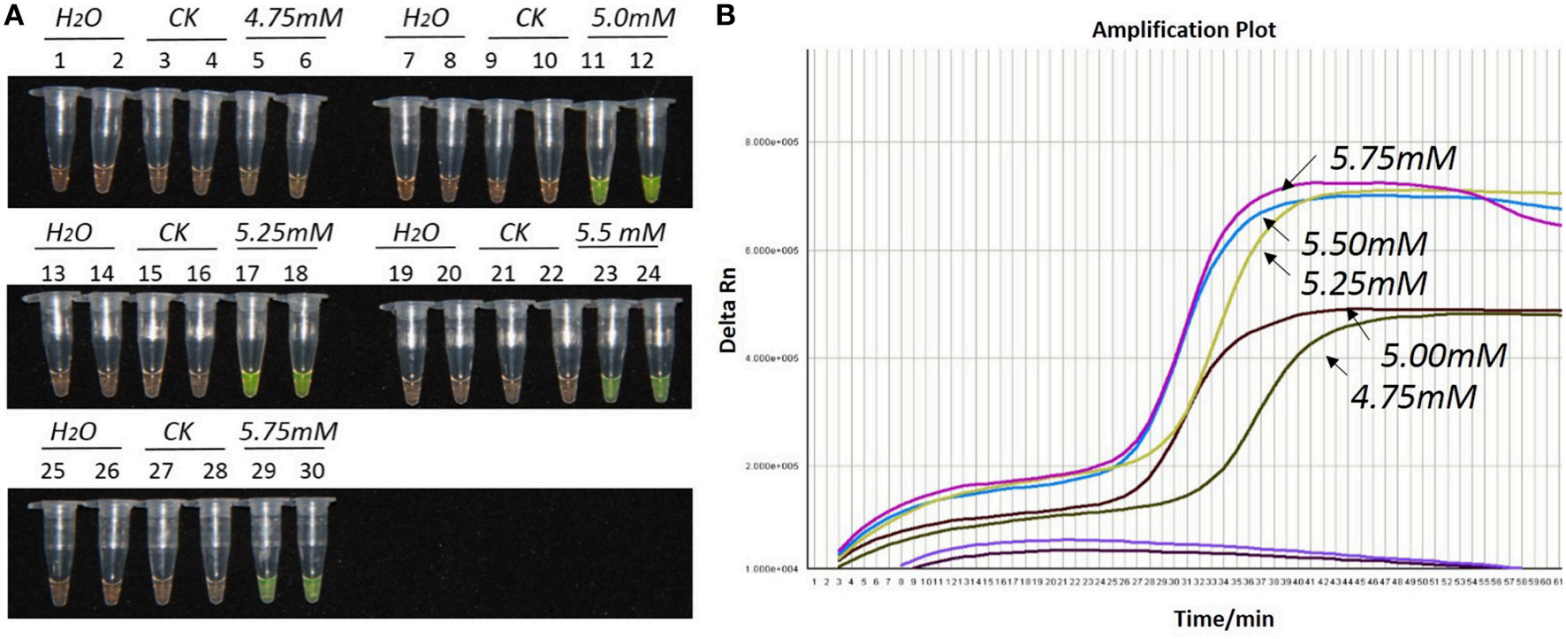
**Optimization of Mg^2+^ concentration for the LAMP reaction of the *bar* transgene. (A)** LAMP products detected by 1000 × SYBR Green I. **(B)** The amplification curves obtained in real-time LAMP based on different Mg^2+^ concentrations. **(A)**: Tubes 1, 2, 7, 8, 13, 14, 19, 20, 25, and 26: ddH_2_O. Tubes 3, 4, 9, 10, 15, 16, 21, 22, 27, and 28: FN95-1702 (negative control, CK). Tubes 5, 6, 11, 12, 17, 18, 23, 24, 29, and 30: the plasmid 1Ac0229. Tubes 1–6, 7–12, 13–18, 19–24, and 25–30: Concentration of Mg^2+^ is 4.75, 5.00, 5.25, 5.50, and 5.75 mM, respectively, two technical replicates. **(B)** Curves separately represent the Mg^2+^ concentrations of 5.75, 5.5, 5.25, 5.00, and 4.75 mM, from left to right. The colored line at the very bottom indicates the blank and negative controls.

**Figure 2:**
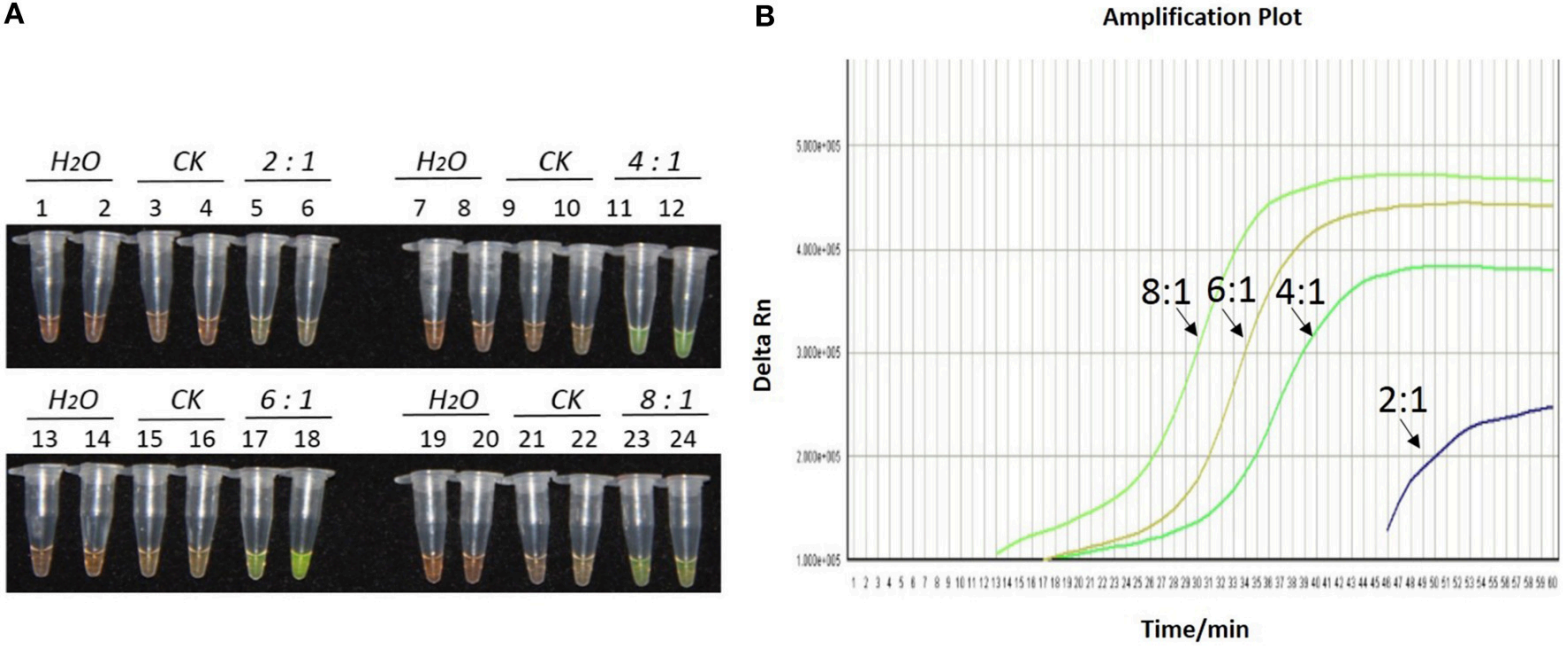
**Optimization of ratios of inner vs. outer primers for the LAMP reaction of the *bar* transgene. (A)** LAMP products detected by 1000 × SYBR Green I. **(B)** The amplification curves obtained in real-time LAMP based on different ratios of inner and outer primers. **(A)** Tubes 1, 2, 7, 8, 13, 14, 19, and 20: ddH_2_O. Tubes 3, 4, 9, 10, 15, 16, 21, and 22: FN95-1702 (negative control, CK). Tubes 5, 6, 11, 12, 17, 18, 23, and 24: the plasmid 1Ac0229. Tubes 1–6, 7–12, 13–18, and 19–24: Ratio of inner and outer primers is 2:1, 4: 1, 6: 1, and 8:1, respectively, two technical replicates. **(B)** Curves separately represent the ratios of inner and outer primers of 8:1, 6: 1, 4: 1, and 2:1, from left to right. The lines representing the blank and negative controls could not be observed at the bottom of this graph.

**Figure 3:**
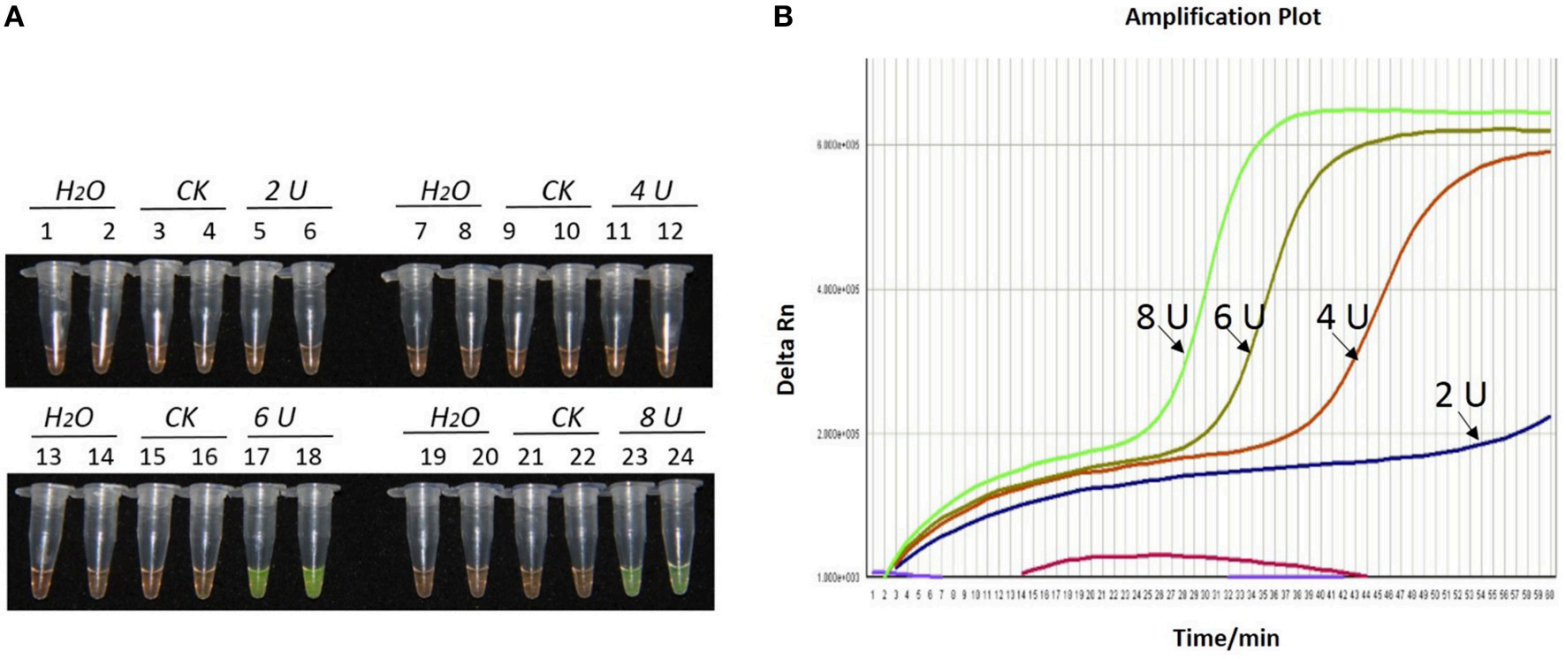
**Optimization of *Bst* DNA polymerase concentration for the LAMP reaction of the *bar* transgene. (A)** LAMP products detected by 1000 × SYBR Green I. **(B)** The amplification curves obtained in real-time LAMP based on different dosage of *Bst* DNA polymerase. **(A)** Tubes 1, 2, 7, 8, 13, 14, 19, and 20: ddH_2_O. Tubes 3, 4, 9, 10, 15, 16, 21, and 22: FN95-1702 (negative control, CK). Tubes 5, 6, 11, 12, 17, 18, 23 and 24: the plasmid 1Ac0229. Tubes 1–6, 7–12, 13–18, and 19–24: *Bst* DNA polymerase concentrations of 2.0, 4.0, 6.0, and 8.0 U, respectively, two repeats. **(B)** Curves separately represent the dosage of *Bst* DNA polymerase of 8.0, 6.0, 4.0, and 2.0 U, from left to right. The colored line at the very bottom indicates the blank and negative controls.

During the optimization of the LAMP system, initial LAMP products were identified by gel extraction and sequencing (data not shown). The effects of Mg^2+^ concentration, *Bst* DNA polymerase amount and concentration ratio between inner and outer primers, were tested (Figures 1–**3**).

As shown in Figures 1A, 2A, 3A, with a concentration of Mg^2+^ between 5.00 and 5.75 mM, a ratio of inner vs. outer primers of 4:1, 6:1, and 8:1, and a dosage of *Bst* DNA polymerase from 6.0 to 8.0 U, the tubes containing template 1Ac0229 with *bar* gene turned yellowish green, while the tubes without plasmid 1Ac0229 remained orange. However, a more intense yellowish-green color was observed with 5.25 mM of Mg^2+^ (Figure 1A), a 6:1 ratio of inner vs. outer primers (Figure 2A) and 6.0 U of *Bst* DNA polymerase (Figure 3A).

The detection results of amplification curves obtained in real-time LAMP showed some J shaped curves in Figures 1B, 2B, 3B. When the concentration of Mg^2+^ was between 5.50 and 5.75 mM, the delta Rn showed a sharp increase in fluorescence from 25 to 35 min, then a platform stage from 40 to 60 min. While the concentration of Mg^2+^ at 5.25, 5.00, and 4.75 mM, the delta Rn rised dramatically from 30 to 40 min, from 28 to 38 min, and from 32 to 40 min, respectively. However, the ultimate delta Rn at the platform stage was different. With concentrations of Mg^2+^ was between 5.25 and 5.75 mM, the ultimate delta Rn was two-fold higher than that of 4.75 and 5.00 mM (Figure 1B). When the ratio of inner to outer primers was 2:1, 4:1, 6:1, and 8:1, the time period of the delta Rn increasing sharply was about 46, 33, 28, and 23 min, respectively. And the ultimate delta Rn at the platform stage was decreased along with lower ratio of inner to outer primers from 8:1 to 2:1 (Figure 2B). Similarly, the amplification improved at 56, 37, 30, and 25 min as the dosage of *Bst* DNA polymerase increased from 2.0 to 8.0 U, and the ultimate delta Rn at the platform stage of the dosage of *Bst* DNA polymerase between 4.0 and 6.0 U was similar, which was stronger than that of 2.0 U (Figure 3B).

The above results show that the optimization of the LAMP reaction can be achieved with minimum costs and time. The final parameters for the reaction were: 5.25 mM of Mg^2+^, a 6:1 ratio of inner to outer primers, and 6.0 U of *Bst* DNA polymerase per reaction.

### Sensitivity of LAMP and conventional PCR

**Figure 4:**
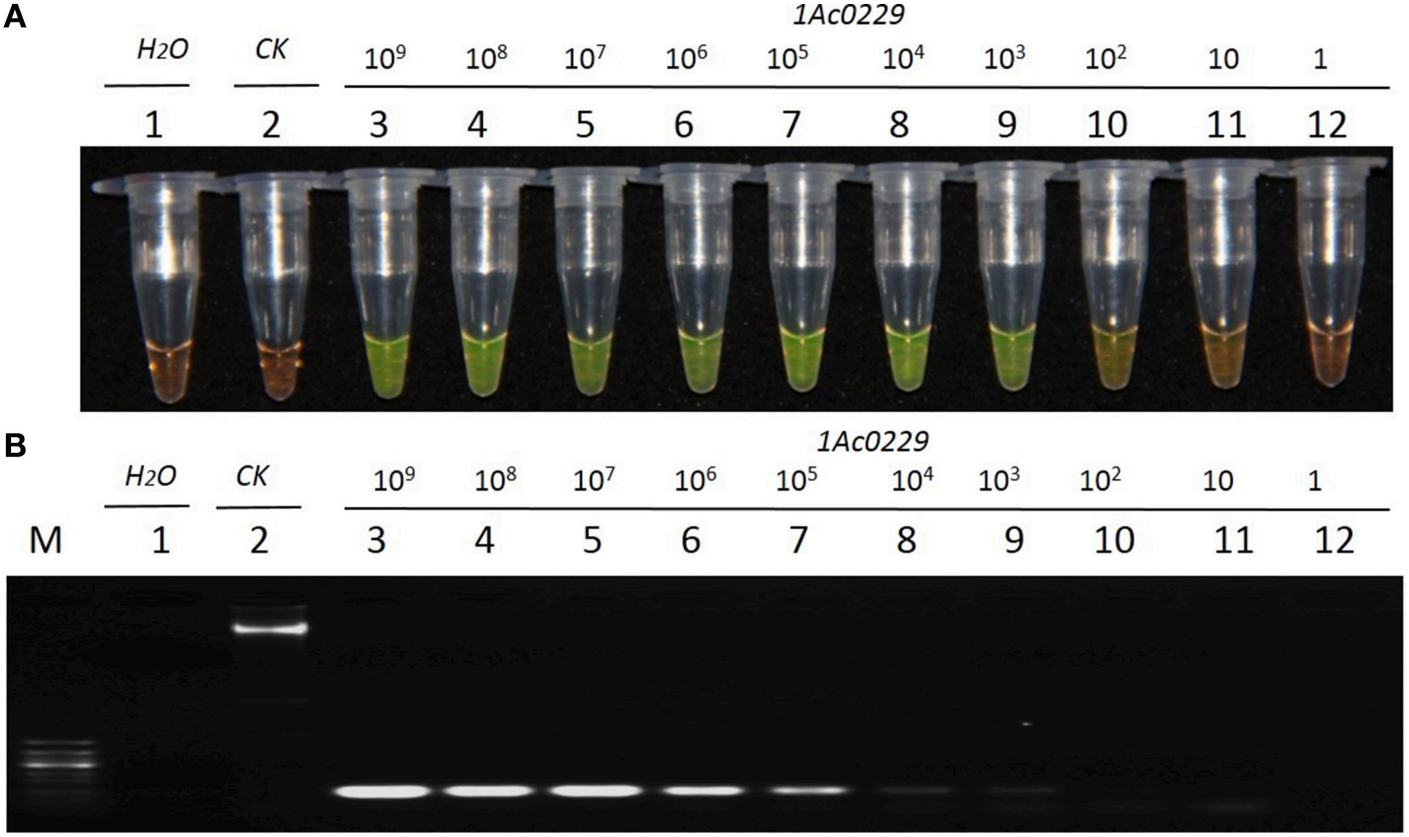
**Sensitivity comparison of the LAMP assay and conventional PCR using the plasmid 1Ac0229 as templates. (A)** LAMP products detected by 1000 × SYBR Green I. **(B)** PCR products detected by agarose gel electrophoresis stained by EB. Tube and lane 1: ddH_2_O. Tube and lane 2: FN95-1702 (negative control, CK). Tubes and lanes 3–12: plasmid 1Ac0229 copies of 1.0 × 10^9^, 1.0 × 10^8^, 1.0 × 10^7^, 1.0 × 10^6^, 1.0 × 10^5^, 1.0 × 10^4^, 1.0 × 10^3^, 1.0 × 10^2^, 1.0 × 10^1^, and 1.0 × 10^0^, respectively. Lane M: 50 bp DNA marker.

The result from Figure 4A revealed that the detection limit of LAMP method was about 1.0 × 10^1^ copies of plasmid, while that of the conventional PCR method was about 1.0 × 10^2^ copies (Figure 4B). It thus suggested that the sensitivity of LAMP was around 10 times higher than that of the conventional PCR when the test template was plasmid 1Ac0229.

### Specificity of LAMP

**Figure 5:**
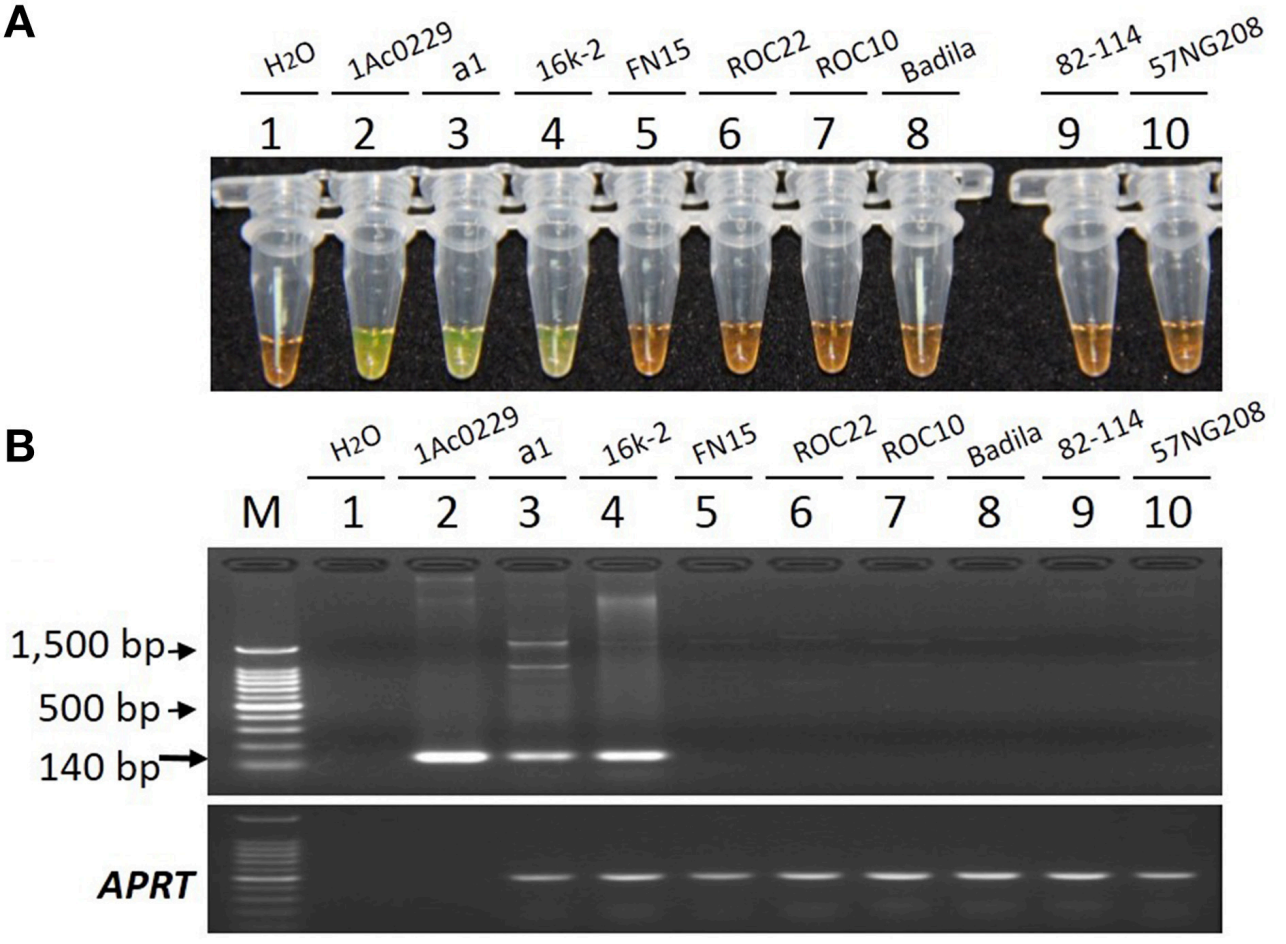
**Specificity comparison of the LAMP assay and conventional PCR. (A)** LAMP products detected by 1000 × SYBR Green I. **(B)** PCR products detected by agarose gel electrophoresis stained by EB. Tube and lane 1: ddH_2_O. Tube and lane 2: the plasmid 1Ac0229 (positive control). Tubes and lanes 3–10: genomic DNA from sugarcane genotypes of a1 (*bar* transgenic line from host cultivar FN15), 16k-2 (*bar* transgenic line from host cultivar ROC22), FN15 (*S*. spp. hybrids), ROC22 (*S*. spp. hybrids), ROC10 (*S*. spp. hybrids), Badila (*S. officinarum*), 82-114 (*S. spontaneum*), 57NG208 (*S. robustum*), respectively. Lane M: 100 bp DNA.

As shown in Figure 5, only the tubes and lanes from the plasmid 1Ac0229, *bar* transgenic sugarcane lines a1 and 16k-2 exhibited positive reactions (Figure 5 tubes and lanes 2–4), while no positive reaction was observed in all the six non-transgenic sugarcane samples, i.e., three *S*. spp. hybrids cultivars FN15, ROC22 and ROC10, one *S. officinarum* Badila, and two wild species: *S. spontaneum* 82-114 and *S. robustum* 57NG208, indicating the developed LAMP system has the same specificity for detecting *bar* transgenic sugarcane.

### Putative *bar* transgenic lines detected by LAMP and conventional PCR

**Figure 6:**
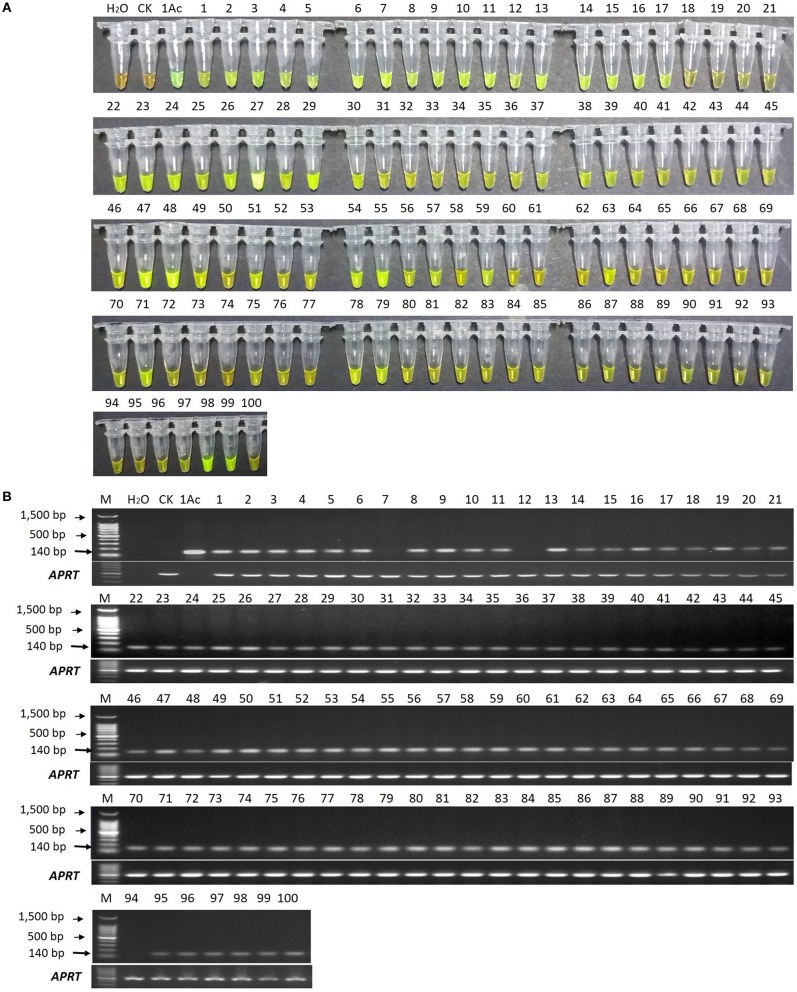
**LAMP and conventional PCR detection results of 100 putative *bar* transgenic sugarcane lines. (A)** LAMP products detected by 1000 × SYBR Green I under white light. **(B)** PCR products detected by agarose gel electrophoresis stained by EB. Tube and lane H_2_O: ddH_2_O. Tube and lane CK: FN95-1702 (negative control). Tube and lane 1Ac: the plasmid 1Ac0229 (positive control). Tubes and Lanes 1–100: the 17 putative *bar* transgenic sugarcane lines in order of p1 ~ p100. Lane M: 100 bp DNA ladder.

In the LAMP assay, orange color reactions were observed in the blank control and negative control (in Figure 6A), while the positive control and all the 100 putative *bar* transgenic lines displayed a yellowish green color, though the lines p18, p19, p20, p21, p72, p73, p74, p94, p95, p96, and p100 (in Figure 6A) were not displayed yellowish color intensely under white light by naked eyes. However, all the positive controls and all the 100 putative *bar* transgenic lines displayed an intense yellowish-green color when the products were detected by 1000 × SYBR Green I under ultraviolet (UV) light, while the blank control and negative control showed a weak yellow color (data not show). These results indicated that all these lines were positive for the *bar* gene.

In the conventional PCR assay, the specific 140 bp product was amplified for all the putative transgenic lines except lines p7, p12, and p94 (Figure 6B).

In order to further confirm the reliability of the LAMP assay in putative *bar* transgenic sugarcane, we used quantitative SYBR Green real-time PCR to determine the copy number of the *bar* gene (results shown in Figure S4 and Table S2) and used quick test strip kit (QuickStix™ Kit for PAT/bar, Envirologix, Inc., USA) to detect bar protein expression in p7, p12, and p94 (shown in Figure S5). The quantitative SYBR Green real-time PCR results showed that all the lines contained the *bar* gene with copy numbers ranging from 0.20 ± 0.00 to 17.91 ± 0.28. Meanwhile, the bar protein expression was detected in all the three lines (p7, p12, and p94) positive in LAMP assay but negative in PCR detection Therefore, the detection results of LAMP, quantitative real-time PCR and protein expression were consistent.

## Discussion

In the present study, for the first time, we developed a sensitive, reliable and rapid visual LAMP detection assay for *bar* transgenic sugarcane. To ensure the robustness, a rigorous control system was always conducted in our protocol containing a negative control (gDNA from non-transgenic parent varieties), a positive control (the plasmid 1Ac0229 with the *bar* gene) and a blank control (ddH_2_O sterilized by filtration with 0.2 μm filter membrane).

Genetic engineering offers an attractive alternative in sugarcane breeding (Gilbert et al., 2005). The *bar* gene, which encodes the enzyme phosphinothricin acetyltransferase (PAT), is usually integrated into sugarcane as (i), a selectable marker gene, (ii), a useful target gene for agronomic trait improvement, or (iii) one of target genes in multi-resistance transgenic sugarcane (Gallo-Meagher and Irvine, 1996; Butterfield et al., 2002). To select the herbicide tolerant sugarcane, the PPT was usually sprayed at the early stage of *in vitro* culture (Gallo-Meagher and Irvine, 1996; Falco et al., 2000). This is a very easy and effective strategy to obtain herbicide tolerant sugarcane (Gallo-Meagher and Irvine, 1996). However, a long time is needed before significant phenotypes appear after PPT spraying (Gallo-Meagher and Irvine, 1996), and thus detection of *bar* transgenic sugarcane in nucleic acid level by PCR or LAMP etc. is necessary, especially for transgenic sugarcane supervision and administration.

Conventional PCR and real-time PCR require expensive and specialized equipment, including PCR thermal cycler or real time quantitative fluorescence thermal cycler, a gel system and a gel scanner, which also raises time cost of detection (about 3–4 h; Zhou et al., 2014; Notomi et al., 2015). On the contrary, LAMP detection is completed in a single step by incubating the LAMP reaction mix (DNA template, primers and a strand displacement DNA polymerase) at a constant temperature (about 65°C) for nearly 1 h (Notomi et al., 2015). In addition to its practical and economical properties, LAMP is suitable for the detection of specific gene such as *cry1Ac* gene in transgenic sugarcane (Zhou et al., 2014).

Here, we firstly optimized the LAMP assay for the following three factors: Mg^2+^ concentration, inner vs. outer primer ratio and *Bst* DNA polymerase dosage. Furthermore, the specificity and sensitivity of the LAMP assay was confirmed.

Previous researches have demonstrated that *Bst* DNA polymerase is a Mg^2+^ dependent enzyme, which utilizes magnesium as a chelate with nucleotidyl di- or tri-phosphates or the NTP substrate and the metal cofactor serves as a mediator of phosphoryl or nucleotidyl transfer (Cowan, 2002). Tomita et al. (2008) found that Mg^2+^ can greatly affect the amplification of LAMP reaction and even resulted in the formation of primer dimers. Notomi et al. (2000) and Tomita et al. (2008) illustrated that low magnesium concentrations may result in extremely low amplification efficiency, whilst excessive magnesium would decrease the specificity of the LAMP reaction. Therefore, Mg^2+^ concentration was assumed to be one of the most important components in LAMP assay and was recommended as the primary factor to be optimized (Notomi et al., 2000, 2015). Here, we concluded that the rational Mg^2+^ concentration range was from 5.00 to 5.75 mM with the optimal being 5.25 mM, which was in accordance with Lee et al. (2009) and Nie (2005).

Appropriate primers are the key factor during LAMP amplification (Notomi et al., 2015). Ingenious design and proper dosage of primers in LAMP system could employ a single strand of DNA shape like a dumbbell with loops at both ends in initial amplification and then continuous sequential progression of the LAMP elongation amplification reaction (Notomi et al., 2015). The availability of primer design software, which contributes to the simplicity of the LAMP technique, could facilitates the generation of appropriate primer sets specific to the input target sequence automatically (Zhou et al., 2014; Liu et al., 2015; Kinoshita et al., 2015). In our study, four primers (FIP, BIP, F3, and B3) and two loop primers (LF and LB), which recognize a total of eight distinct regions on the target *bar* gene, were designed through the online software http://primerexplorer.jp/e/. An optimal dosage ratio (6:1) of inner vs. outer primer (Figure 2) definitely distinguished the negative and positive controls.

Another key factor influencing the LAMP assay is the dosage of the *Bst* DNA polymerase (Guan et al., 2010). With the optimized conditions of Mg^2+^ and ratio of inner vs. outer primers in the present study, we found that *Bst* DNA polymerase dosage ranging from 6.0 to 8.0 U showed good results in LAMP. However, Zhou et al. (2014) revealed that it can even get a positive result when the *Bst* DNA polymerase concentration was as low as 2.0 U when detecting the transgenic *cry1Ac* sugarcane.

Due to the fact that the reaction time of LAMP can be affected by the size of the target DNA sequence, this study utilized a 1 h reaction time for the LAMP assay in accordance to the size of our amplified target DNA sequence (180 bp; Zhou et al., 2014). However, in this study, the real-time LAMP showed that the delta Rn reached into a platform stage was reduced to less than 1 h (Figures 1B, 2B, 3B). This is probably because the loop primers (LF and LB) could reduce the amplification time from 1 h to around 30–40 min (Figures 1B, 2B, 3B).

Previous studies showed that the specificity and sensitivity of LAMP technology were higher than those of the conventional PCR. Wang et al. (2009) investigated that the sensitivity of LAMP was 10 times higher than that of the conventional PCR for the detection of *cry1Ac* transgenic *Oryza sativa*. Shen et al. (2015) found the sensitivity of LAMP was 10 times higher than that of the conventional PCR for detecting *cry2Ab2* and *cp4-epsps* in transgenic corn, cotton, eggplant and soybean. In the present study, no amplicon was detected in the blank and negative controls, thus confirming the specificity of LAMP primers. And the specificity evaluation was further carried out and confirmed on two *bar* transgenic lines and six non-transgenic sugarcane lines with different genetic background: three *S*. spp. hybrids cultivars FN15, ROC22 and ROC10, one *S. officinarum* Badila, and two wild species of *S. spontaneum* 82-114 and *S. robustum* 57NG208. The evaluation results revealed that LAMP assays are high specific to screen transgenic sugarcane employing *bar* gene. Sensitivity analysis showed that the developed LAMP system provided a detection limit of 10 times higher than that of the conventional PCR targeting *bar* gene in transgenic sugarcane.

However, the high sensitivity of LAMP can sometimes lead to false-positive amplification due to cross contamination, caused especially by aerosol in the assay process (Wang et al., 2009). Some product detection methods that involve process of opening the reaction tube such as the AGE which adds SYBR Green I after reaction, are easy to produce aerosols (Wang et al., 2009; Zhou et al., 2014). Therefore, the closure of reaction tube caps, addition of SYBR Green I into the cap center before reaction, or addition of calcein and Mn^2+^, or Hydroxy naphthol blue (HNB) or berberine is recommended (Goto et al., 2009; Fischbach et al., 2015). Here, in order to avoid aerosols, none of LAMP products were analyzed by electrophoresis to detect whether the presence of ladder-like DNA amplification products or not. Many studies used a real-time turbidimeter for the LAMP reaction confirmation (Goto et al., 2009), despite it being expensive for its single application to detect the turbidity of LAMP product. In the case of labs conducting real-time PCR, this technique can be employed during the development of the LAMP assay (Randhawa et al., 2013). Given the high costs of real-time PCR, real-time LAMP was used in the present study only as a supplementary method to develop and optimize the visual LAMP for detecting *bar* transgenic sugarcane.

Conventional PCR is widely adopted to detect the *bar* transgenic sugarcane (Gallo-Meagher and Irvine, 1996; Falco et al., 2000; Butterfield et al., 2002). In this study, using a series of dilutions of plasmid DNA, the developed LAMP method had around 10-fold higher sensitivity than that of the conventional PCR. Results from the detection of putative *bar* transgenic sugarcane lines showed that most putative transgenic lines showed a 140 bp amplicon by PCR technique (97 out of 100, Figure 6B), while all the putative transgenic lines displayed yellowish-green in the LAMP assay (Figure 6A). There are also three transgenic lines (p7, p12, and p94) being *bar* transgenic positive detected in LAMP assay while escaped detection from conventional PCR assay, of which the most probably reason is the lower sensitivity of conventional PCR than that of LAMP. When the amount of each template (1.0 μL of 50ng/μL gDNA) is the same, the bar-transgenic lines with the lower copies (i.e., p7, p12, and p94) maybe below the limit of detection (LOD) of conventional PCR, while above the LOD of LAMP. Besides, we think that these three events (p7, p12, and p94) are *bar*-transgenic sugarcane plants with very low copy number of foreign gene. These lines showed less than one copy of the transgene per cell when detected by qPCR (Table S2), which may represent chimeric lines.

In conclusion, a visual LAMP assay is developed for detection of the *bar* transgenic sugarcane. The specificity is confirmed on two *bar* transgenic sugarcane lines and six non-transgenic sugarcane lines with different genetic background, and the sensitivity evaluation displays the limit of the recombinant plasmid 1Ac0229 being as 10 copies in the LAMP reaction, which is 10 times higher than that of the conventional PCR, indicating the developed LAMP assays is more sensitive than the conventional PCR. The LAMP assay developed here would facilitate *bar*-specific screening to check for GM sugarcane events, as well as to monitor *bar*-specific GM contamination in the field and the commercialization of *bar*-transgenic sugarcane in future.

## Author contributions

Conceived and designed the experiments: DZ, LX, YQ. Performed the experiments: DZ, CW, ZL, WL, SG, JG, YS. Analyzed the data: DZ, CW, ZL, YC, WL, YS. Wrote the paper: DZ, SG, JG, LX, YQ. Revised and approved the final version of the paper: LX, YQ, DZ.

### Conflict of interest statement

The authors declare that the research was conducted in the absence of any commercial or financial relationships that could be construed as a potential conflict of interest.

## Supplementary material

Figure S1 **Construction of the plasmid pGcry1Ac0229 (1Ac0229)**.

Figure S2 **Agarose gel electrophoresis of the plasmid 1Ac0229 PCR products**. Lane M: 100 bp DNA ladder. Lanes 1: ddH_2_O (blank control). Lanes 2: FN15 PCR products (negative control). Lanes 3: the plasmid 1Ac0229 PCR products.

Figure S3 **LAMP primers and their positions in *bar* gene (GenBank: EU048867.1) sequence**.

Figure S4 **The amplification curves and standard curves obtained by quantitative SYBR Green real-time PCR with the primer pair of *bar* gene**. In the performed quantitative SYBR Green real-time PCR assays, the standard curve formula is *y* = −3.354 x + 36.898, coefficient of determination (*R*^2^ = 0.999) and amplification efficiency (*E* = 0.99).

Figure S5 **Bar protein expression in putative *bar* transgenic sugarcane by quick test strip kit detection**.

Table S1 **The sequence information of LAMP primers used in this experiment**.

Table S2 **Estimation of copy number of *bar* gene in transgenic sugarcane by quantitative SYBR Green real-time PCR**.
