# Supplementary material for: Detection of Bar Transgenic Sugarcane with a Rapid and Visual Loop-Mediated Isothermal Amplification Assay
Source: Front Plant Sci. 2016 Mar 8;7:279. doi: 10.3389/fpls.2016.00279 (PMC4782128; doi:10.3389/fpls.2016.00279)
Supplement: Figure S1 — Construction of the plasmid pGcry1Ac0229 (1Ac0229). [file DataSheet1.pdf]

Supplementary Material

Detection of *bar* transgenic sugarcane with a rapid and visual loop-mediated isothermal amplification assay

Dinggang Zhou, Chunfeng Wang, Zhu Li, Yun Chen, Shiwu Gao, Jinlong Guo, Wenying Lu, Yachun Su, Liping Xu*, Youxiong Que*

Key Laboratory of Sugarcane Biology and Genetic Breeding, Fujian Agriculture and Forestry University, Ministry of Agriculture, Fuzhou, Fujian, China

***Correspondence:** Liping Xu and Youxiong Que, Key Laboratory of Sugarcane Biology and Genetic Breeding, Fujian Agriculture and Forestry University, Ministry of Agriculture, Fuzhou 350002, China. E-mail: xlpmail@126.com, queyouxiong@126.com

1. Supplementary Figures and Tables

1.1 Supplementary Figures

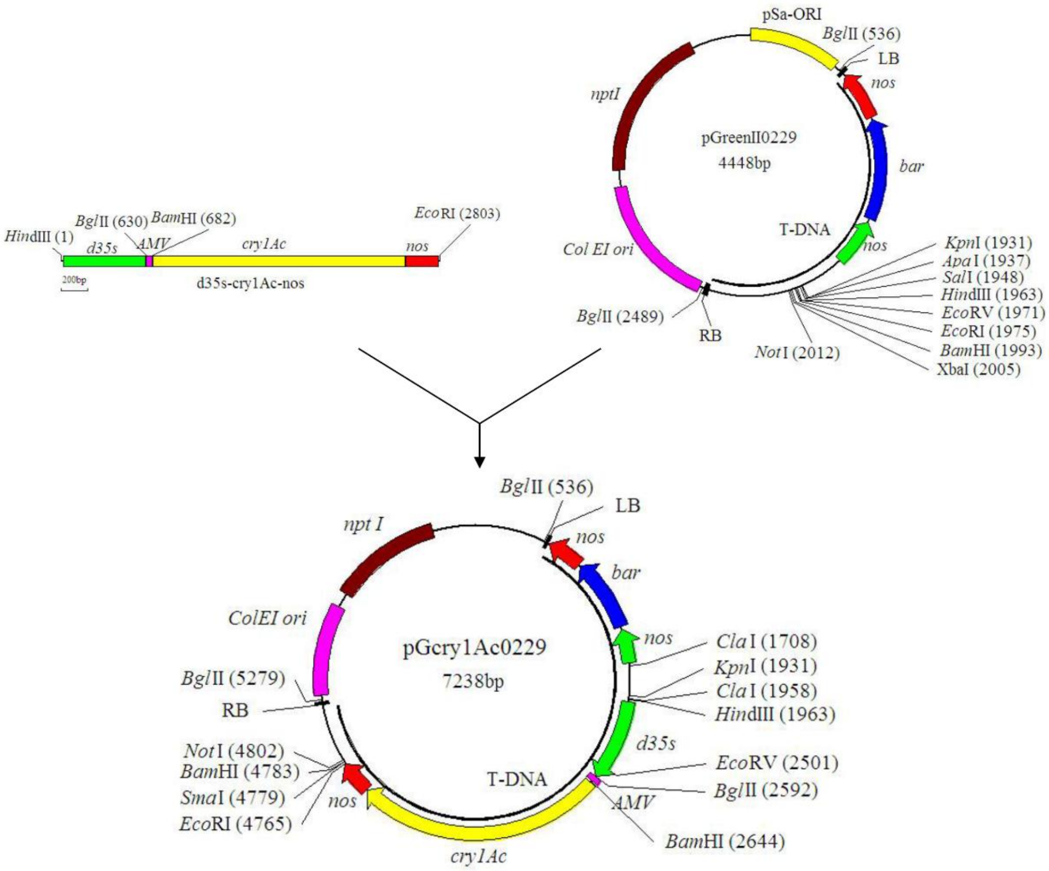

Figure S1. Construction of the plasimid pGcry1Ac0229 (1Ac0229).

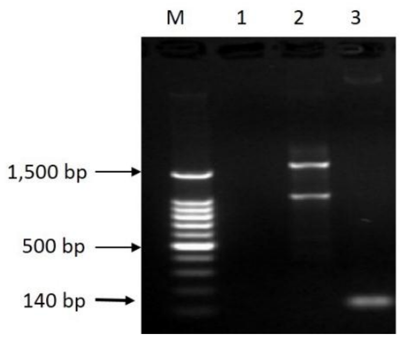

Figure S2 Agarose gel electrophoresis of the plasmid 1Ac0229 PCR products. Lane M: 100 bp DNA ladder. Lanes 1: ddH_2O (blank control). Lanes 2: FN15 PCR products (negative control). Lanes 3: the plasmid 1Ac0229 PCR products.

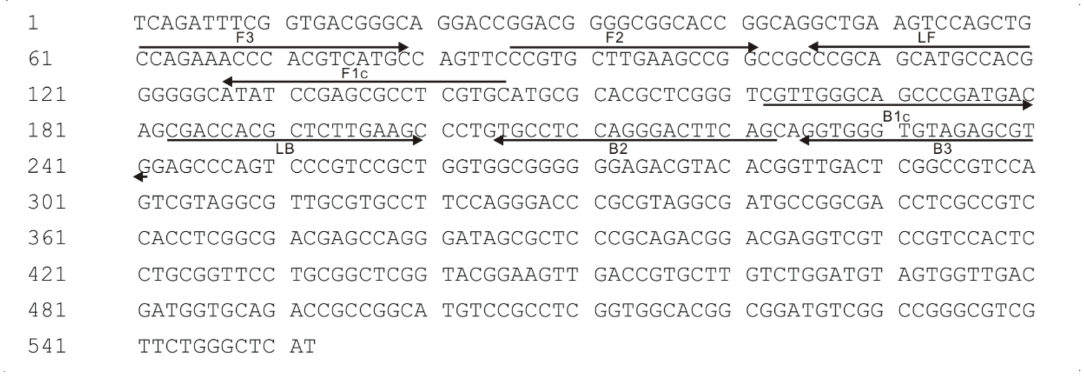

Figure S3. LAMP primers and their positions in *bar* gene (GenBank: EU048867.1) sequence.

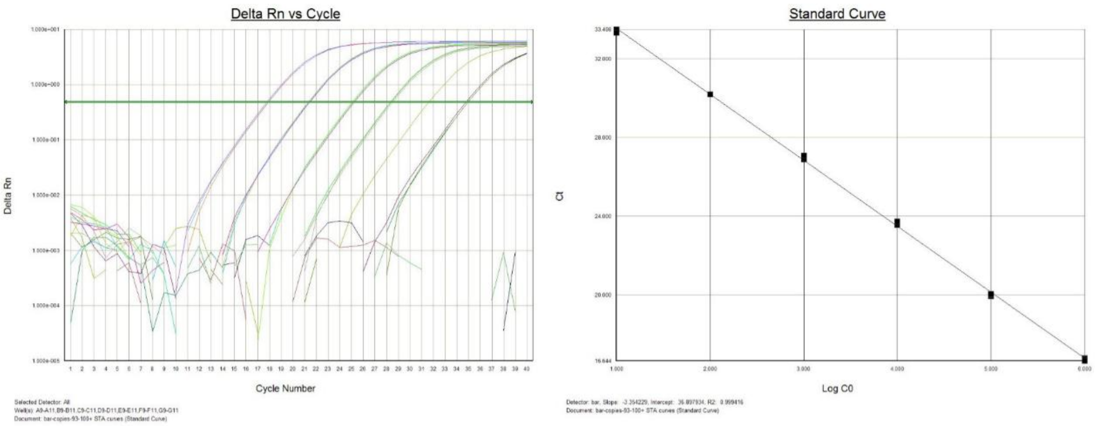

Figure S4. The amplification curves and standard curves obtained by quantitative SYBR Green real-time PCR with the primer pair of *bar* gene. In the performed quantitative SYBR Green real-time PCR assays, the standard curve formula is y=-3.354 x + 36.898, coefficient of determination (R^2=0.999) and amplification efficiency (E= 0.99).

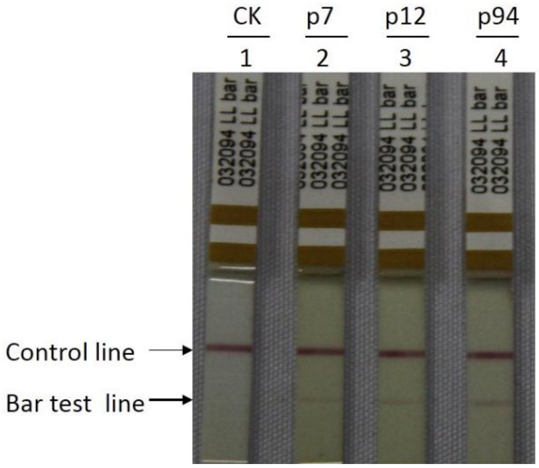

Figure S5. Bar protein expression in putative *bar* transgenic sugarcane by quick test strip kit detection.

1.2 Supplementary Tables

Table S1. The sequence information of LAMP primers used in this experiment.

Primer	Position	Primer sequences (5'- 3')
F3	61-79	CCAGAAACCCACGTCATGC
B3	224-241	CACGCTCTACACCCACCT
FIP (F1c+F2)	127-145<br>86-101	GCACGAGGCGCTCGGATAT-<br>CCGTGCTTGAAGCCGG
BIP (B1c+B2)	162-180<br>205-222	CGTTGGGCAGCCCGATGAC-<br>CTGAAGTCCCTGGAGGCA
LF	106-120	CGTGGCATGCTGCGG
LB	183-200	CGACCACGCTCTTGAAGC

Table S2. Estimation of copy number of *bar* gene in transgenic sugarcane by quantitative SYBR Green real-time PCR.

Lines	Copy number of *bar* in single cell (Mean ± St)	Lines	Copy number of *bar* in single cell (Mean ± St)	Lines	Copy number of *bar* in single cell (Mean ± St)
p1	1.82±0.04	p35	7.39±0.54	p69	6.99±0.25
p2	1.15±0.02	p36	7.25±0.57	p70	6.49±0.15
p3	1.94±0.02	p37	4.82±0.12	p71	7.47±0.17
p4	3.41±0.11	p38	7.13±0.09	p72	5.99±0.11
p5	4.16±0.09	p39	5.86±0.09	p73	8.23±0.17
p6	2.82±0.07	p40	8.25±0.19	p74	7.33±0.33
p7	0.35±0.00	p41	7.20±0.10	p75	7.98±0.03
p8	1.83±0.06	p42	8.47±0.77	p76	8.04±0.20
p9	1.57±0.04	p43	7.37±0.51	p77	9.07±0.12
p10	1.49±0.01	p44	8.94±0.75	p78	5.50±0.09
p11	0.56±0.00	p45	7.77±0.16	p79	14.15±0.17
p12	0.20±0.00	p46	8.90±0.11	p80	11.81±0.15
p13	5.89±0.08	p47	6.63±0.20	p81	13.18±0.04
p14	1.29±0.12	p48	6.06±0.02	p82	18.54±0.11
p15	1.34±0.02	p49	8.34±0.17	p83	13.67±0.50
p16	1.90±0.01	p50	9.24±0.17	p84	16.72±0.55
p17	0.71±0.03	p51	6.98±0.16	p85	11.83±0.11
p18	0.83±0.06	p52	6.76±0.25	p86	11.17±0.05
p19	8.75±0.36	p53	10.08±0.22	p87	13.08±0.25
p20	0.65±0.01	p54	9.01±0.12	p88	11.62±0.10
p21	4.30±0.08	p55	5.49±0.11	p89	8.58±0.42
p22	3.60±0.05	p56	6.22±0.28	p90	11.44±0.28
p23	4.09±0.08	p57	7.57±0.23	p91	13.84±0.50
p24	3.91±0.07	p58	7.74±0.40	p92	16.90±0.39
p25	7.88±0.25	p59	9.78±0.29	p93	17.31±0.81
p26	4.72±0.36	p60	8.89±0.51	p94	0.53±0.01
p27	4.40±0.13	p61	9.41±0.85	p95	17.01±0.75
p28	3.52±0.13	p62	9.94±0.17	p96	17.91±0.28
p29	3.35±0.03	p63	7.17±0.12	p97	16.36±0.73
p30	2.74±0.07	p64	9.51±0.34	p98	12.38±0.20
p31	4.41±0.17	p65	10.29±0.33	p99	14.27±0.64
p32	10.33±0.13	p66	8.45±0.48	p100	13.70±0.12
p33	11.52±0.18	p67	7.10±0.46	FN15(CK)	0.02±0.00
p34	9.68±0.40	p68	5.51±0.17		
